# Icariin Treatment Protects Against Gentamicin-Induced Ototoxicity via Activation of the AMPK-SIRT3 Pathway

**DOI:** 10.3389/fphar.2021.620741

**Published:** 2021-02-18

**Authors:** Yue Hu, Xiulan Ma

**Affiliations:** Department of Otolaryngology Head and Neck Surgery, Shengjing Hospital of China Medical University, Shenyang, China

**Keywords:** ototoxicity, aminoglycosides, icariin, AMP-activated protein kinase, reactive oxygen species

## Abstract

Ototoxicity is a serious health problem that greatly affects millions of people worldwide. This condition is caused by the entry of aminoglycosides into auditory hair cells, subsequently inducing reactive oxygen species (ROS) production and accumulation. Several strategies have been adopted to overcome irreversible ROS-induced hair cell loss in mammals. In recent years, icariin, a major active component of the traditional herb Epimedium, has been widely studied and revealed to have antioxidant, anti-inflammatory, and anti-apoptotic properties. In this study, we found that icariin pretreatment improved the survival rate of gentamicin-treated House Ear Institute-Organ of Corti 1 (HEI-OC1) cells and cochlear explants. Icariin remarkably suppressed HEI-OC1 cell apoptosis and inhibited ROS production in cells. Notably, icariin upregulated PGC-1α (SIRT3 promoter) and SIRT3 expression in HEI-OC1 cells. In addition, SIRT3 inhibition significantly attenuated the anti-apoptotic effect of icariin. We also found that icariin can increase AMPK phosphorylation. Further studies showed that inhibition of SIRT3 activity had no significant effect on AMPK phosphorylation. Furthermore, the AMPK inhibitor compound C significantly suppressed SIRT3 expression, meaning that AMPK, as an upstream molecule, regulates SIRT3 expression. Meanwhile, inhibition of AMPK activity significantly reduced the protective effect of icariin on gentamicin ototoxicity. Based on these results, icariin exerts its protective effect on gentamicin-induced ototoxicity via activation of the AMPK-SIRT3 signaling pathway, thus providing a new strategy for treating ototoxicity caused by aminoglycoside antibiotics.

## Introduction

Aminoglycoside antibiotics are currently the most widely used antibiotics worldwide (Van Boeckel et al., 2014), largely due to their low price and remarkable antibacterial properties. However, the incidence of ototoxicity is high during clinical treatment (3.2–47%), and consequent hearing damage is permanent (Xie et al., 2011). This has placed a heavy burden on the lives of millions of people worldwide and an economic burden on society (O’Sullivan et al., 2017). Aminoglycoside antibiotics mainly cause hearing loss by damaging the hair cells on the basement membrane of the inner ear. For decades, studies of hair cell regeneration through gene editing have achieved remarkable results; however, the new hair cells were non-functional, and their survival time was very short (Chen et al., 2019b). Therefore, we have focused on developing new drugs to alleviate ototoxicity caused by aminoglycoside antibiotics. After aminoglycosides enter the hair cells, large amounts of intracellular reactive oxygen species (ROS) are produced and excessively deposited, resulting in the oxidation of DNA, lipid, protein, and other important cell components (Leis et al., 2015; Esterberg et al., 2016; Jiang et al., 2017). Icariin, the flavonoid glucoside from herbs in the genus Epimedium, has various beneficial pharmacological and biological properties, including neuroprotective (Zeng et al., 2019), antitumor (Wu et al., 2015), anti-inflammatory, and antioxidant effects (Wu et al., 2012; Sun et al., 2019). Icariin can ameliorate cisplatin-induced cytotoxicity in human embryonic kidney 293 cells by suppressing ROS production (Zhou et al., 2019) and protect osteoblasts against iron overload by reducing ROS levels (Jing et al., 2019). Thus, it is of great interest whether the antioxidant effect of icariin can also be used to prevent gentamicin-induced ototoxicity.

This study was conducted to develop a potential drug that inhibits the ototoxicity of aminoglycoside antibiotics and to provide a new target for the diagnosis and treatment of drug-induced deafness. We performed cell viability analysis, a terminal deoxynucleotidyl transferase dUTP nick end labeling (TUNEL) assay, western blotting, and an immunofluorescence assay to determine the effect of icariin on gentamicin-treated House Ear Institute-Organ of Corti 1 (HEI-OC1) cells and cochlear explants. SIR2 is a family of histone deacetylases, which are widely distributed in mammalian cells and have multiple functions (Meng et al., 2018). SIRT3, SIRT4, and SIRT5 have been found in mitochondria. Among them, SIRT3 is closely related to oxidative stress and has been shown to be closely related to inner ear hair cell damage. SIRT3 plays a key role during the process of ROS removal (Brown et al., 2014; Han et al., 2020). AMPK has been confirmed to be involved in the pathophysiological process of aminoglycoside antibiotic-induced ototoxicity, and phosphorylated AMPK is downregulated in gentamicin-treated hair cells (Ebnoether et al., 2017). Moreover, SIRT3 has been reported as a downstream target of AMPK in some disease models (Meng et al., 2018). The activation of the AMPK-SIRT3 signaling pathway contributes to the improvement of mitochondrial function and thus reduces disease progression. However, in hair cells, the role of AMPK-SIRT3 signaling pathway activation in the protective effect of icariin against gentamicin-induced ototoxicity is still unknown. In our study, the protective effect of icariin against gentamicin-induced ototoxicity was determined for the first time. Furthermore, we also demonstrated the important role of the AMPK-SIRT3 signaling pathway in this protective effect of icariin against gentamicin-induced ototoxicity.

## Materials and Methods

### Ethics Declaration

This study was approved by the Institutional Animal Care and Use Committee of the China Medical University. All applicable institutional guidelines for the care and use of animals were followed.

### Cell Culture and Drug Treatment

HEI-OC1 cells were cultured in Dulbecco’s modified Eagle’s medium (DMEM) containing 10% fetal bovine serum (Bioind, Beit-Haemek, Israel) at 33°C in a 10% CO_2_ incubator. The cultured HEI-OC1 cells were treated with 5, 50, 500, and 1,000 μM gentamicin (No. MB1331-S, Meilun, Dalian, China) for 12 h to establish an ototoxic model. To determine the protective effect of icariin on gentamicin-induced ototoxicity, HEI-OC1 cells were pretreated with 0.2, 2, or 20 μM icariin (No. MB2189, Meilun) for 24 h before gentamicin was added. To identify the role of the AMPK-SIRT3 signaling pathway in the ability of icariin to protect against gentamicin-induced ototoxicity, we divided the cultured cochlear explants into seven groups: Group 1, no treatment; Group 2, gentamicin treatment for 12 h; Group 3, icariin treatment for 24 h and gentamicin treatment for 12 h; Group 4, compound C (No. S7840, 10 μM Selleck, United States) treatment for 2 h, icariin treatment for 24 h, and gentamicin treatment for 12 h; Group 5, 3-(1H-1,2,3-triazol-4-yl) pyridine (3-TYP, No. abs814581, 50 μM, Absin Biotechnology, Shanghai, China) treatment for 2 h, icariin treatment for 24 h, and gentamicin treatment for 12 h; Group 6, compound C treatment for 38 h; and Group 7, 3-TYP treatment for 38 h. Icariin, compound C and 3-TYP stock were dissolved in dimethyl sulfoxide (DMSO) (No. D8371, Solarbio, Beijing, China) and diluted with culture medium (Final concentration of 0.1% DMSO).

### Cochlear Explants Dissection

Kunming mice (Changsheng, Liaoning, China) were used as the model for this study, which belongs to the Swiss mice. On postnatal day 3, Kunming mice were anesthetized and sacrificed by decapitation. The cochlear explants were dissected and cultured in DMEM-F12.

### Western Blotting

The cultured HEI-OC1 cells were washed three times with phosphate-buffered saline (PBS) and placed on ice with cold RIPA buffer (Millipore, Billerica, MA, United States) for 30 min. The protein content was determined using a bicinchoninic acid protein assay kit (Beyotime, Shanghai, China). The protein was transferred onto a polyvinylidene fluoride membrane for SDS-PAGE. The membrane was sealed with 5% bovine serum albumin for 2 h and incubated overnight at 4°C with primary antibodies. After washing three times with Tris-buffered saline containing Tween 20, the membrane was incubated with the secondary antibody (1:10,000; Absin) at room temperature (24°C) for 2 h. An ECL Kit (Affinity Biosciences, Cincinnati, OH, United States) was used to visualize the protein signals. The following antibodies were used: Rabbit anti-Bcl-2 (No. ab182858 1:1,000, Abcam, United States), rabbit anti-Bax (No. ab32503, 1:1,000, Abcam), rabbit anti-caspase-3 (No. ab13847, 1:1,000, Abcam), rabbit anti-SIRT3 (No. ab86671, 1:1,000, Abcam), rabbit anti-PGC-1α (No. AF5395, 1:1,000, Affinity), rabbit anti-β-Tubulin (No. ab18207, 1:1,000, Abcam), rabbit anti-p-AMPK (No. ab133448, 1:3,000, Abcam), and AMPK (No. ab32047, 1:3,000, Abcam). First photograph of Western Blot can be found in Supplementary Figures 4-6.

### TUNEL Assay

The cultured HEI-OC1 cells were washed once with PBS and fixed with 4% paraformaldehyde for 30 min. PBS containing 0.3% TritonX-100 was then added, and the cells were incubated at room temperature for 5 min. After washing twice with PBS, 50 μL TUNEL detection solution (No. C1089, Beyotime) was added to each well and incubated at 37°C in the dark for 60 min. The film was sealed with anti fluorescence quenching solution (No. P0128S, Beyotime) after washing three times with PBS, and the cells were observed under a fluorescence microscope. Cells from 10 visual fields (same location in the three slides) per sample were counted and averaged. The collected data were analyzed using GraphPad Prism software (GraphPad, v. 8.0.2, San Diego, CA, United States).

### ROS Detection

DCFH-DA working solution (No. MB4682, Meilun) was used to detect active oxygen production in HEI-OC1 cells. Cells were incubated at 33°C for 60 min, washed twice with PBS, and then analyzed by flow cytometry (BD Biosciences, San Jose, CA, United States) to measure ROS levels. ROS levels were also measured by immunofluorescence, after incubation with DCFH-DA solution (No. MB4682, Meilun), the cells were observed under fluorescence microscope. The cochlear explants were incubated with DCFH-DA working solution (Meilun) for 60 min, washed three times with PBS, and then analyzed using an LSM 880 confocal microscope (Zeiss, Oberkochen, Germany).

### Quantitative Real-Time PCR Experiments

Total RNA was extracted from HEI-OC1 cells per group using TRIzol reagent (Tiangen Biotech Co., Ltd., Beijing, China). First-strand cDNA was synthesized using reverse transcriptase (Tiangen Biotech), and qRT-PCR was performed using SuperReal PreMix Plus kit (Tiangen Biotech) on an ABI7500 Real-time PCR system (Applied Biosystems, Foster City, CA, United States). The forward and reverse primers used in this study were synthesized by Takara Bio (Shiga Japan) and had the following sequences: GAPDH, 5′ GTA​TGA​CTC​CAC​TCA​CGG-3′, and 5′ GGT​CTG​GCT​CCT​GGA​AGA-3′; Bcl-2, 5′-ATC​GCC​CTG​TGG​ATG​ACT​G-3′, and 5′ GCC​AGG​AGA​AAT​CAA​ACA​GAG​GC-3′; BAX, 5′ GCC​AGG​AGA​AAT​CAA​ACA​GAG​GC-3′, and 5′ TGT​GTC​CAC​GGC​GGC​AAT​CAT​C-3′; Caspase-3, 5′ GGA​AGC​GAA​TCA​ATG​GAC​TCT​GG-3′, and 5′ GCA​TCG​ACA​TCT​GTA​CCA​GAC​C-3′.

### Cell Viability Assay

The cultured HEI-OC1 cells were collected and counted using Cellometer Auto T4 (Nexcelom Bioscience LLC, Lawrence, MA, United States), with the concentration adjusted to 2.0 × 10^5^ cells/ml. The cells were seeded into 96-well plates (100 μL/well) and cultured overnight to allow attachment to the bottom of the wells. After treatment at 33°C for 12 h, cell viability was evaluated in a TACS® MTT cell proliferation assay (Trevigen, Gaithersburg, MD, United States). The absorbance was measured using a Spectra Max 5 Plate Reader and Soft Max Pro 5.2 software (Molecular Devices Corp., Sunnyvale, CA, United States). The average OD of the control cells was considered to be equivalent to a 100% survival rate.

### Immunofluorescence Assay

After culture, the cochlear explants were fixed with 4% paraformaldehyde for 15 min, washed with PBS three times for 5 min each, and subjected to immunofluorescence staining. PBT-1 (0.1% TritonX-100, No. MB2486 and 5% donkey serum, No. MB4516 were purchased from Meilun Biology, and 1% bovine serum albumin in 1× PBS, No. abs9157 was purchased from Absin Biotechnology) was used to block the cochlear explants for 1 h. The explants were then incubated with myosin-VIIa antibody (1:500; Proteus Biosciences, Ramona, CA, United States) overnight at 4°C. Alexa Fluor® 488 secondary antibodies (1:200; Abcam) were used to count the number of hair cells. Photographs were acquired with an LSM 880 confocal microscope (Zeiss).

### Statistical Analysis

The data were tested for normality using the Kolmogorov-Smirnov test and quantile-quantile plot using SPSS software (SPSS, Inc., Chicago, IL, United States). Data are presented as the means ± SD from three independent experiments. A two-tailed, unpaired Student’s t-test was performed to analyze significant differences. *p*-values < 0.05 were considered to indicate statistical significance.

## Results

### Icariin Pretreatment Enhances HEI-OC1 Cell Viability

**FIGURE 1:**
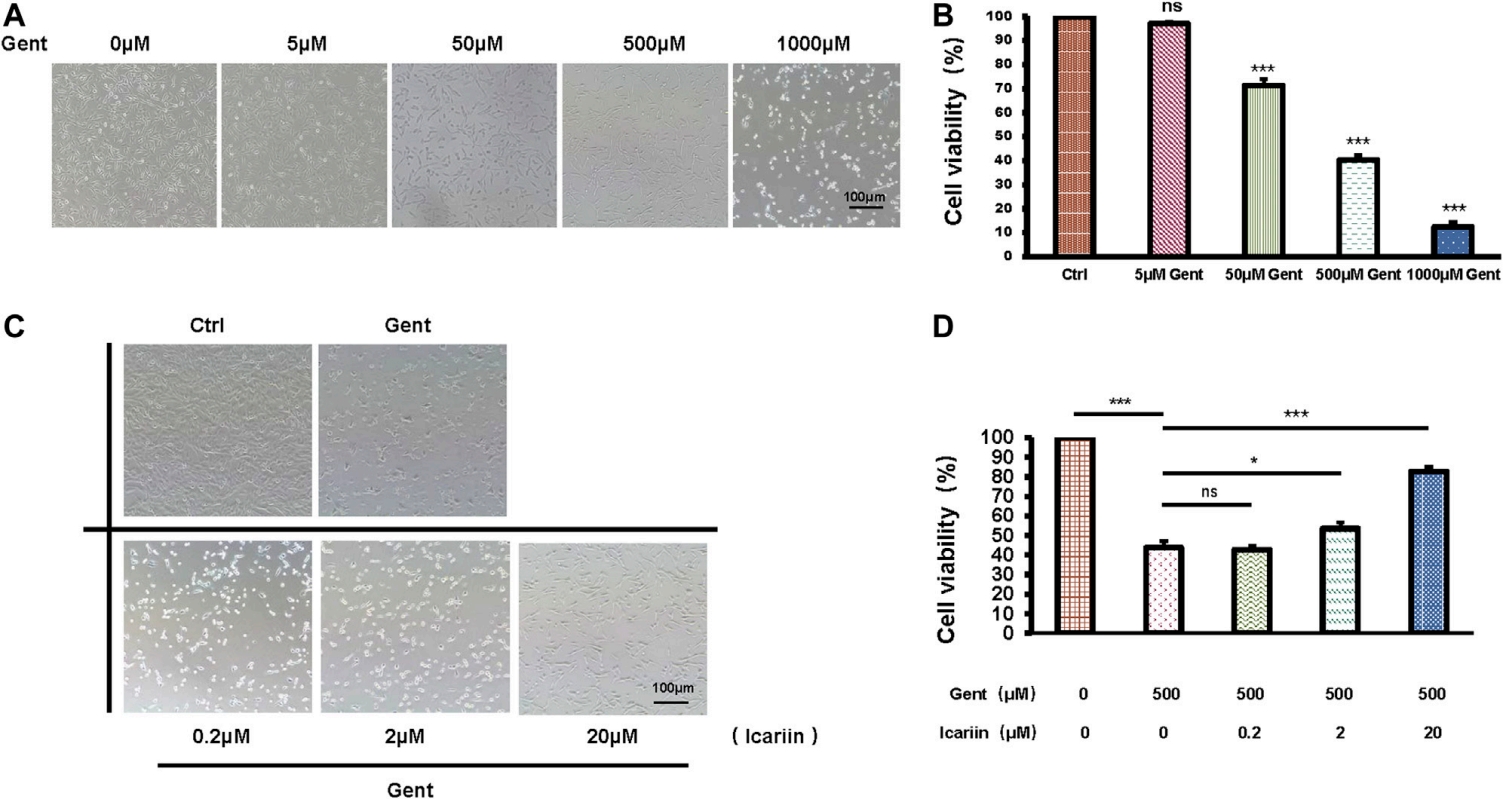
Icariin improves the survival rate of gentamicin-treated HEI-OC1 cells. **(A)** HEI-OC1 cells were treated with increasing concentrations of gentamicin (0, 5, 50, 500, and 1,000 μM) for 12 h. The survival of HEI-OC1 cells significantly decreased following treatment with 500 μM gentamicin. **(B)** Quantification of cell viability. Scale bar = 100 μM. **(C)** HEI-OC1 cells were pretreated with increasing concentrations of icariin (0.2, 2, and 20 μM). Cell viability was significantly increased following pretreatment with high concentrations of icariin. **(D)** Quantification of cell viability. Scale bar = 100 μM. Each experiment was performed three times. Ns, not significant. Scale bar = 100 μM; N = 8 in each group; *p < 0.05; ***p < 0.001; one-way analysis of variance and Tukey’s multiple comparison test.

After treatment with increasing concentrations of gentamicin (5, 50, 500, and 1,000 μM) for 12 h, the mean survival rate of HEI-OC1 cells was significantly lower in the 500 μM group (40.34 ± 2.05%) than in the control group (100%), confirming that an ototoxicity model was successfully established (Figures 1A,B). Thus, we used 500 μM as the working concentration of gentamicin for subsequent experiments. After pretreatment with icariin (0.2, 2, and 20 μM) for 24 h, the cell numbers and viability of HEI-OC1 cells were significantly increased in a dose-dependent manner (82.72 ± 2.37% vs. 43.71 ± 3.35%; Figures 1C,D). We carried out DMSO's control groups to exclude its pharmacological effect on HEI-OC1 cells (Supplementary Figures 8A,B).

### Icariin Inhibits Gentamicin-Induced Apoptosis in HEI-OC1 Cells

**FIGURE 2:**
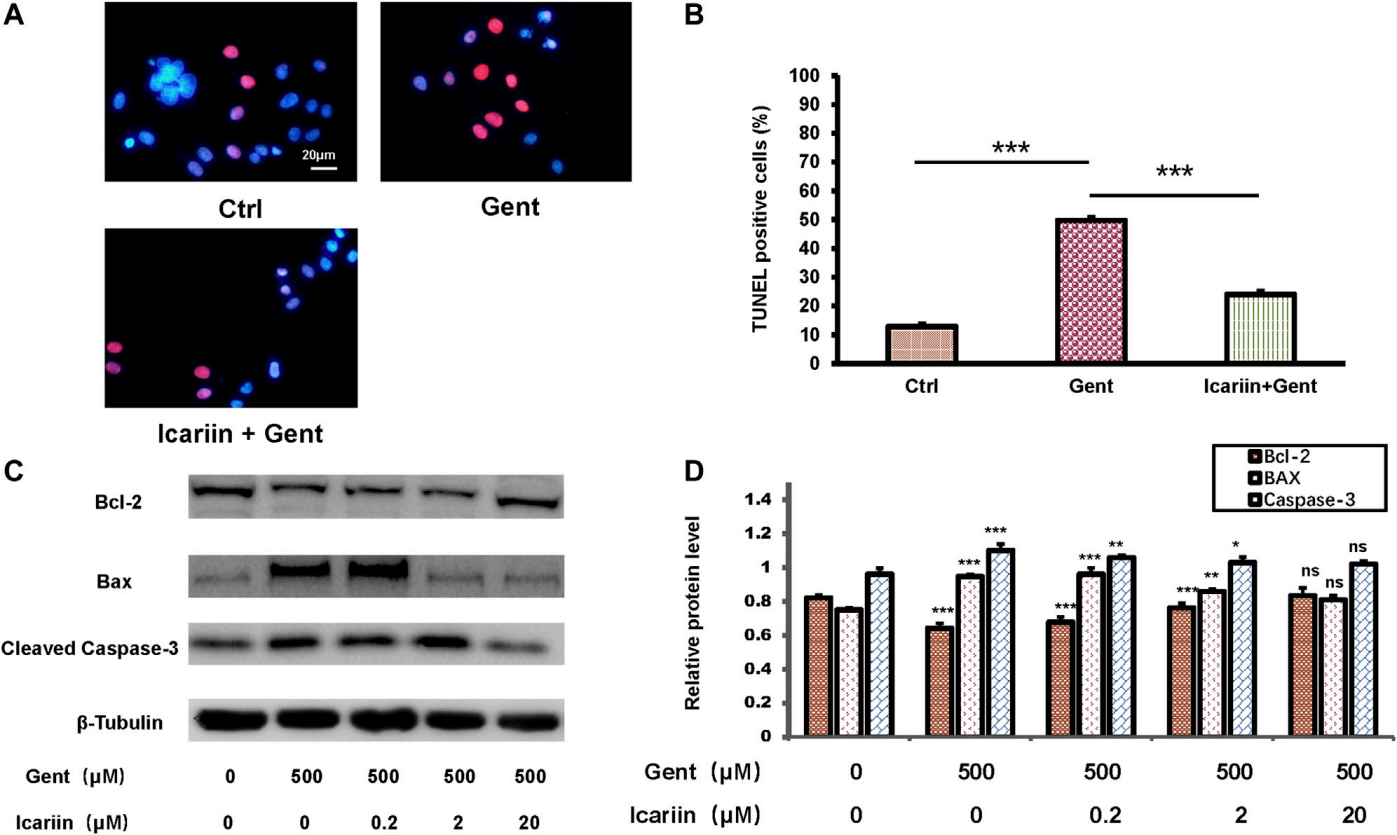
Icariin inhibits gentamicin-induced apoptosis in HEI-OC1 cells. **(A)** HEI-OC1 cells were divided into three groups: control, gentamicin, and icariin + gentamicin. The number of TUNEL-positive cells was distinctly lower in the icariin + gentamicin group than in the other groups. Scale bar = 20 μM **(B)** Measurement of levels of TUNEL-positive cells in **(A)**. **(C)** Expression changes of the apoptotic markers Bcl-2, Bax, and Caspase-3 were tested with and without icariin pretreatment. β-Tubulin was used as a loading control. **(D)** Western blot analyses of Bcl-2, Bax, and Caspase-2 proteins with or without icariin pretreatment. Each experiment was performed three times. Ns, not significant; N = 6 in each group; *p < 0.05; ***p < 0.001; one-way analysis of variance and Tukey’s multiple comparison test.

Though the role of apoptosis was proved previously, we used q-PCR experiments to confirm gentamicin’s effect on HEI-OC1 cells (Supplementary Figures 1A-C). To determine the effect of icariin upon gentamicin-induced apoptosis, HEI-OC1 cells were divided into the control group, 500 μM gentamicin treatment group, and 500 μM gentamicin treatment group pretreated with 20 μM icariin (Figures 2A,B). Compared to the control, the number of TUNEL-positive cells was significantly decreased in the gentamicin-treated group pretreated with icariin (24.92 ± 2.02% vs. 50.81 ± 2.04%). Furthermore, groups pretreated with icariin showed reduced Bax (0.81 ± 0.03 vs. 0.95 ± 0.01) and cleaved caspase-3 (1.02 ± 0.02 vs. 1.10 ± 0.04) protein expression and increased Bcl-2 (0.83 ± 0.04 vs. 0.64 ± 0.03) protein expression in a dose-dependent manner (Figures 2C,D).

### Icariin Inhibits Intracellular ROS Production in HEI-OC1 Cells

**FIGURE 3:**
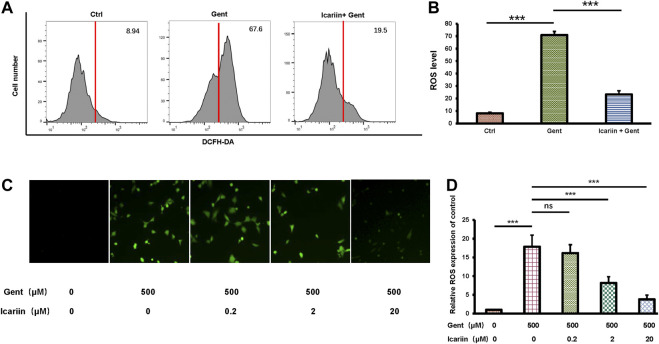
Icariin inhibits production of intracellular reactive oxygen species (ROS). **(A)** Intracellular ROS production was examined by flow cytometry. Cells were divided into three groups: control, 500 μM gentamicin, and 20 μM icariin + 500 μM gentamicin. Gentamicin-induced elevation of ROS levels was significantly attenuated by icariin pretreatment. **(B)** Measurement of ROS levels in **(A)**. **(C)** Intracellular ROS production was observed by immunofluorescence. Cells were pretreated with increasing concentrations of icariin before gentamicin was added. ROS levels significantly decreased with icariin pretreatment. Scale bar = 100 μM. **(D)** Measurement of ROS levels in **(C)**. Each experiment was performed three times. Ns, not significant; N = 6 in each group; *p < 0.05; ***p < 0.001; one-way analysis of variance and Tukey’s multiple comparison test.

To determine the anti-ROS effect of icariin, we carried out both flow cytometry analysis and immunofluorescence. The same control and experimental groups were used as previously described (Figures 3A,B). Flow cytometry analysis showed that icariin pretreatment successfully reduced intracellular ROS levels in gentamicin-treated HEI-OC1 cells (23.33 ± 2.91% vs. 70.82 ± 2.90%) (Figures 3C,D). Accordingly, we also used DCFH-DA to stain for intracellular ROS production. The results showed that as the concentration of icariin increased, the level of ROS significantly decreased (3.77 ± 1.15 vs. 17.87 ± 3.07). Icariin also showed anti-ROS effect upon gentamicin-ototoxicity in cochlear explants (Supplementary Figure 2).

### Icariin Promoted SIRT3 Activity and Expression and Activated the AMPK Signaling Pathway

**FIGURE 4:**
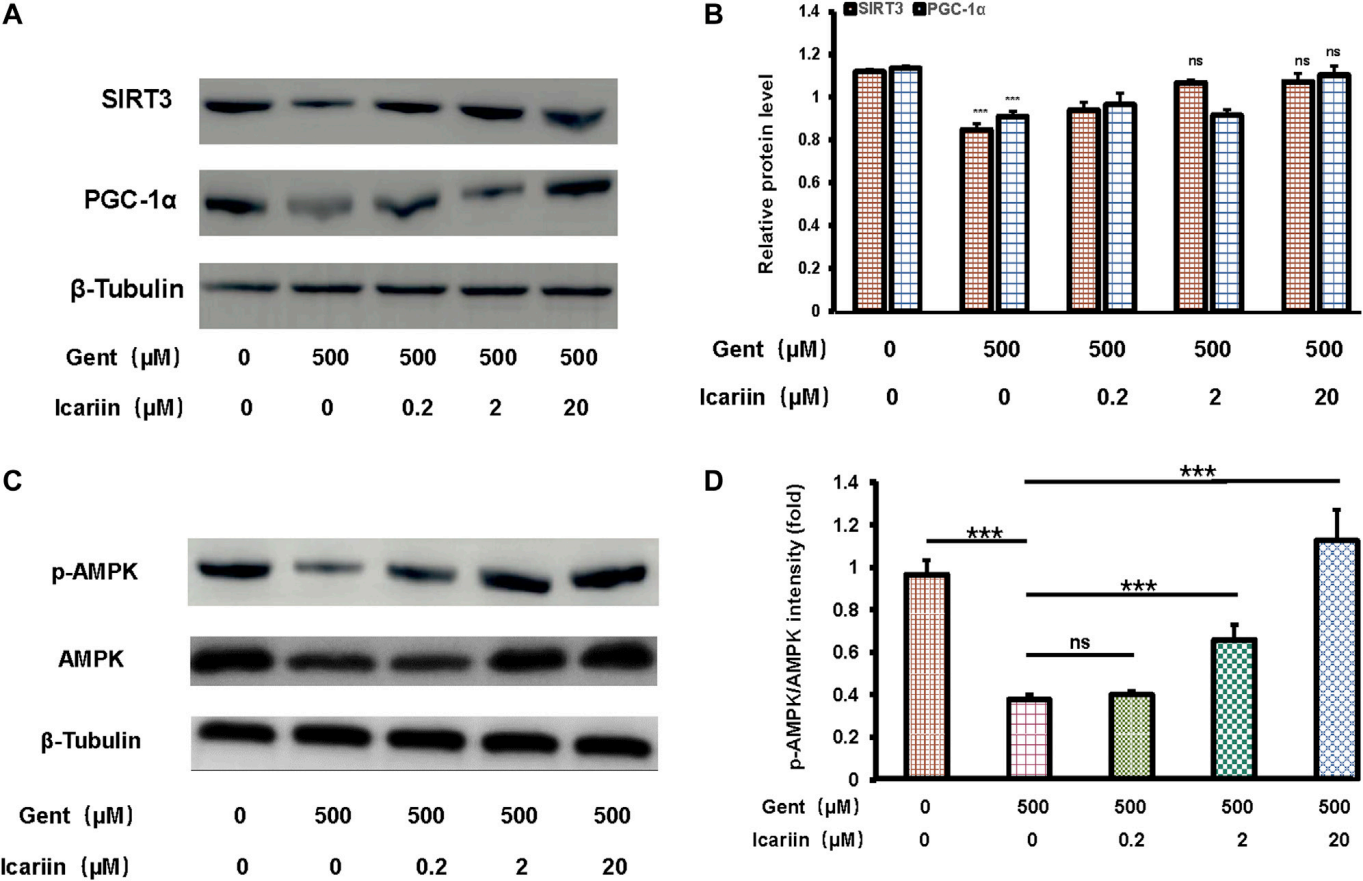
Icariin enhanced SIRT3 expression and promoted AMPK phosphorylation activity. **(A)** We divided cells into five groups: control; 500 μM gentamicin treatment for 12 h; 0.2 μM icariin pretreatment for 24 h then 500 μM gentamicin treatment for 12 h; 2 μM icariin pretreatment for 24 h then 500 μM gentamicin treatment for 12 h; and 20 μM icariin pretreatment for 24 h then 500 μM gentamicin treatment for 12 h. After collecting cells, PGC-1α and SIRT3 expression was measured by western blotting. **(B)** Quantification of PGC-1α and SIRT3 protein expression. β-Tubulin was used as a loading control. **(C)** Western blot analyses of p-AMPK and AMPK in HEI-OC1 cells. Experimental groups are divided as previously described. β-Tubulin was used as a loading control. **(D)** Quantification of AMPK phosphorylation. Each experiment was performed three times. Ns, not significant; N = 6 in each group; *p < 0.05; ***p < 0.001; one-way analysis of variance and Tukey’s multiple comparison test.

Icariin pretreatment significantly promoted SIRT3 (1.07 ± 0.04 vs. 0.85 ± 0.03) and PGC-1α (1.10 ± 0.04 vs. 0.91 ± 0.03) expression, suggesting that SIRT3 is involved in the protective effect of icariin against gentamicin-induced ototoxicity (Figures 4A,B). Further, when gentamicin-damaged HEI-OC1 cells were pretreated with increasing concentrations of icariin, the protein expression of phosphorylated AMPK increased significantly (1.13 ± 0.14 vs. 0.38 ± 0.02; Figures 4C,D).

### SIRT3 and the AMPK Signaling Pathway are Involved in the Protective Effect of Icariin Against Gentamicin-Induced HEI-OC1 Cell Damage

**FIGURE 5:**
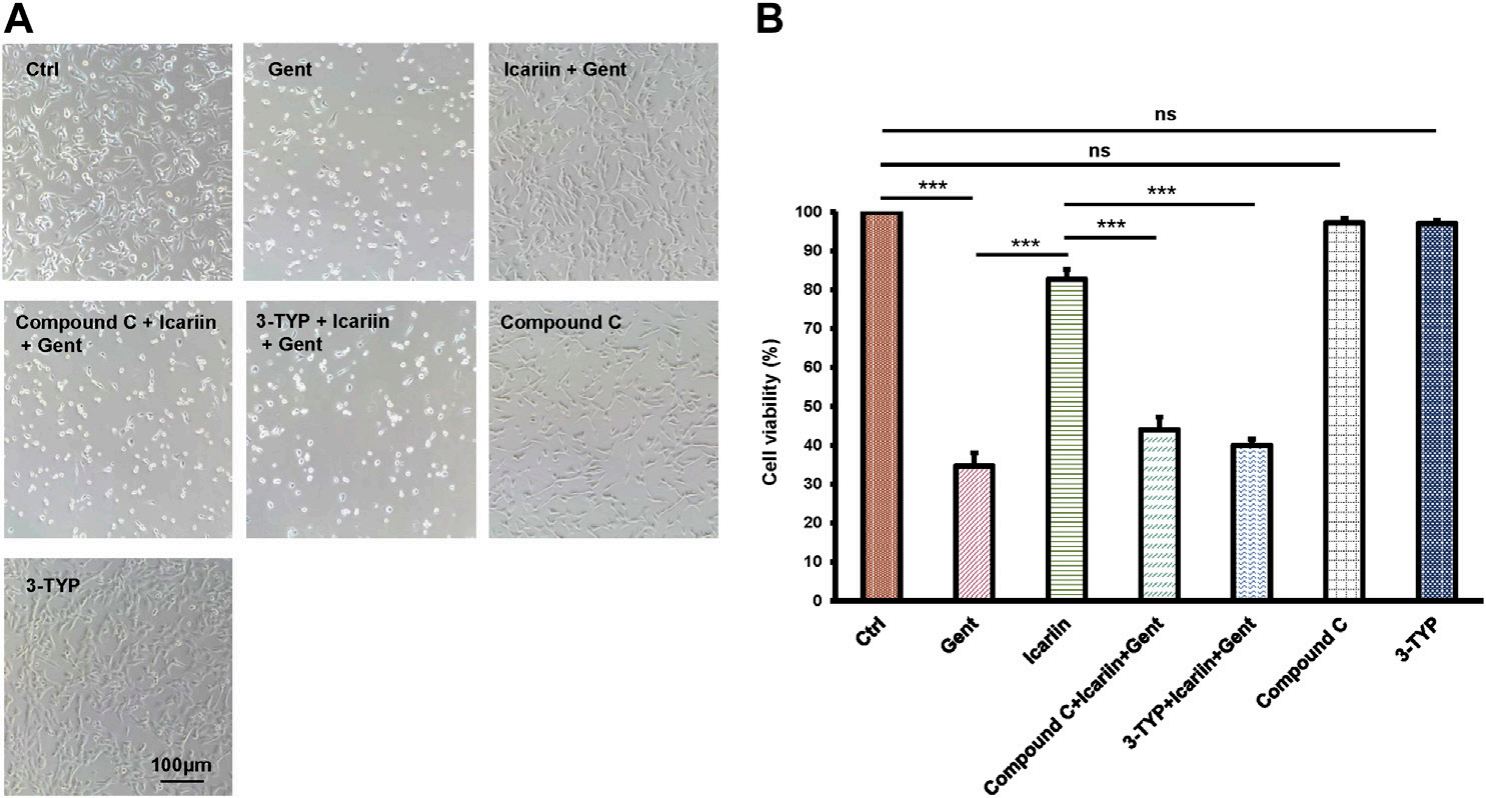
Inhibition of SIRT3 activity and the AMPK signaling pathway decreased HEI-OC1 cell viability with icariin pretreatment. **(A)** To determine the role of SIRT3 and AMPK in the protective effect of icariin on gentamicin-damaged HEI-OC1 cells, cells were pretreated with 50 μM 3-TYP (SIRT3 inhibitor) and 10 μM compound C (AMPK inhibitor) for 2 h. Cell viability in the inhibitor group was significantly lower compared with that in the non-inhibitor group. In addition, cell viability was not significantly affected by single inhibitor treatment. **(B)** Measurement of cell viability in **(A)**. Each experiment was performed three times. Ns, not significant; N = 8 in each group; *p < 0.05; ***p < 0.001; one-way analysis of variance and Tukey’s multiple comparison test.

**FIGURE 6:**
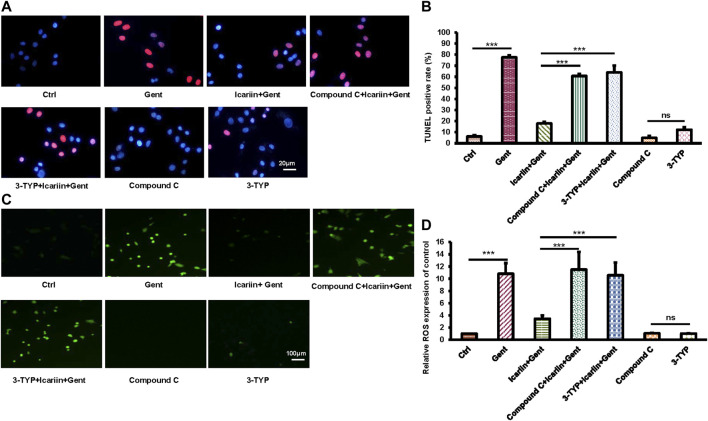
Inhibition of SIRT3 activity and AMPK signaling pathway attenuated the anti-apoptotic and anti-oxidative stress functions of icariin. **(A)** We divided HEI-OC1 cells into seven groups: control, 500 μM gentamicin treatment for 12 h; 200 μM icariin pretreatment for 24 h then 500 μM gentamicin treatment for 12 h; 10 μM compound C treatment for 2 h, icariin treatment for 24 h, and then gentamicin treatment for 12 h; 50 μM 3-TYP treatment for 2 h, icariin treatment for 24 h, and then gentamicin treatment for 12 h; 10 μM compound C treatment for 38 h; and 50 μM 3-TYP treatment for 38 h. TUNEL staining was used to determine the apoptosis rate of HEI-OC1 cells. Scale bar = 20 μM. **(B)** Measurement of the amount of TUNEL-positive cells in **(A)**. **(C)** Similar experimental groups to those previously described were used. The level of ROS in HEI-OC1 cells was determined by DCFH-DA staining. Scale bar = 100 μM. **(D)** Measurement of ROS levels in cells in **(C)**. Each experiment was performed three times. Ns, not significant; N = 6 in each group; *p < 0.05; ***p < 0.001; one-way analysis of variance and Tukey’s multiple comparison test.

In order to further explore the role of SIRT3 and the AMPK signaling pathway in the protective effect of icariin against gentamicin-induced ototoxicity in HEI-OC1 cells, we added the SIRT3 inhibitor 3-TYP and the AMPK inhibitor compound C before icariin treatment (Figures 5A,B). Working concentration of 3-TYP and compound C was determined firstly (Supplementary Figure 7). The results of the cell viability assay showed that the addition of inhibitors reversed the protective effect of icariin (3-TYP: 40.32% ± 1.63% vs. 82.7% ± 2.49%; Compound C: 38.27% ± 3.27% vs. 82.7% ± 2.49%). The viability of HEI-OC1 cells with single inhibitor (3-TYP or compound C) treated along did not significantly change. In our previous study, icariin was confirmed to suppress gentamicin-induced HEI-OC1 cell apoptosis and inhibit intercellular ROS production. We also confirmed the role of SIRT3 and the AMPK signaling pathway in the resistance of icariin to gentamicin-induced apoptosis and endogenous ROS production (Figures 6A,B). Cell apoptosis was marked with TUNEL staining. When cells were pretreated with 3-TYP and compound C before icariin was added, cell apoptosis was significantly increased (3-TYP: 63.91% ± 6.30% vs. 17.87% ± 1.26%; Compound C: 60.76% ± 1.87 vs. 17.87% ± 1.26%) (Figures 6C,D). Endogenous ROS production was measured by DCFH-DA staining, and both inhibitors reversed the effect of icariin on antioxidative stress (3-TYP: 10.57 ± 2.08 vs. 3.45 ± 0.55; Compound C: 11.49 ± 2.91).

### SIRT3 and the AMPK Signaling Pathway are Involved in the Protective Effect of Icariin Against Gentamicin-Induced Cochlear Explant Damage

**FIGURE 7:**
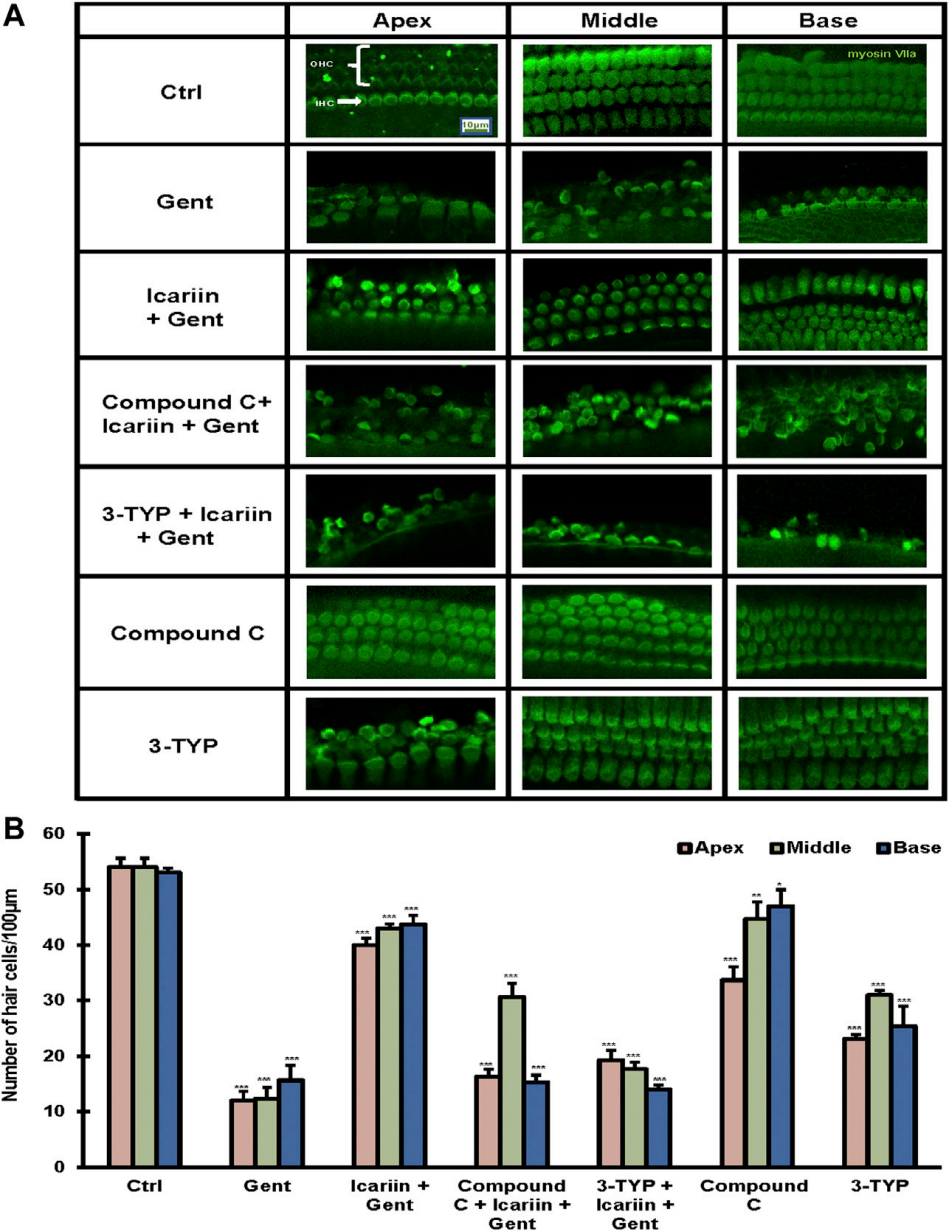
SIRT3 and AMPK signaling pathway are involved in the protective effect of icariin on gentamicin-damaged cochlear explants. **(A)** Cochlear explants were dissected from P3 Kunming mice. We divided the cochlear explants into seven groups, control, 500 μM gentamicin treatment for 12 h; 200 μM icariin pretreatment for 24 h then 500 μM gentamicin treatment for 12 h; 10 μM compound C treatment for 2 h, icariin treatment for 24 h, and then gentamicin treatment for 12 h; 50 μM 3-TYP treatment for 2 h, icariin treatment for 24 h, and then gentamicin treatment for 12 h; 10 μM compound C treatment for 38 h; and 50 μM 3-TYP treatment for 38 h. We divided the cochlear tissue cultured *in vitro* into three parts: base, middle, and apex. Hair cells were labeled with the classic hair cell marker Myosin VIIa (green). Scale bar = 10 μM **(B)** Quantitative analysis of the number of hair cells in the Organ of Corti. Each experiment was performed three times. Ns, not significant; N = 4 in each group; *p < 0.05; ***p < 0.001; one-way analysis of variance and Tukey’s multiple comparison test.

To further confirm our findings, the cochlea tissue was isolated from Kunming mice, and cultured in DMEM F-12 medium. Ototoxicity induced by gentamicin in cochlear explants has been determined previously (Supplementary Figure 3). First, we determined the protective effect of icariin upon gentamicin-induced cochlear explant damage. We divided the cochlear explants into three groups: control, 500 μM gentamicin, and 20 μM icariin and 500 μM gentamicin. By myosin VII a-labeled immunofluorescence detection, we found that gentamicin treatment reduced the number of hair cells. The damage to three lines of our hair cells (OHC) was more severe than that to inner hair cells (IHC), and icariin significantly improved the survival rate of hair cells. In order to explore the role of SIRT3 and the AMPK signaling pathway in the protective effect of icariin against gentamicin-induced cochlear explant injury, we added two additional groups. These two groups received 3-TYP (SIRT3 inhibitor) and compound C (AMPK inhibitor) for 2 h before icariin treatment. Notably, the addition of inhibitors weakened the protective effect of icariin on cochlear explants. In addition, to exclude the influence of the two inhibitors themselves on the stock status of cochlear explants, we added two single inhibitor treatment groups. The results showed that the single inhibitor did not significantly change the survival status of cochlear explants (Figures 7A,B). These results suggest that SIRT3 and the AMPK signaling pathway play an important role in the protective effect of icariin against gentamicin ototoxicity. We added DMSO control groups to exclude DMSO's pharmacological effect upon cells (Supplementary Figures 9A,B).

### The AMPK Pathway Regulates SIRT3 Expression in HEI-OC1 Cells

**FIGURE 8:**
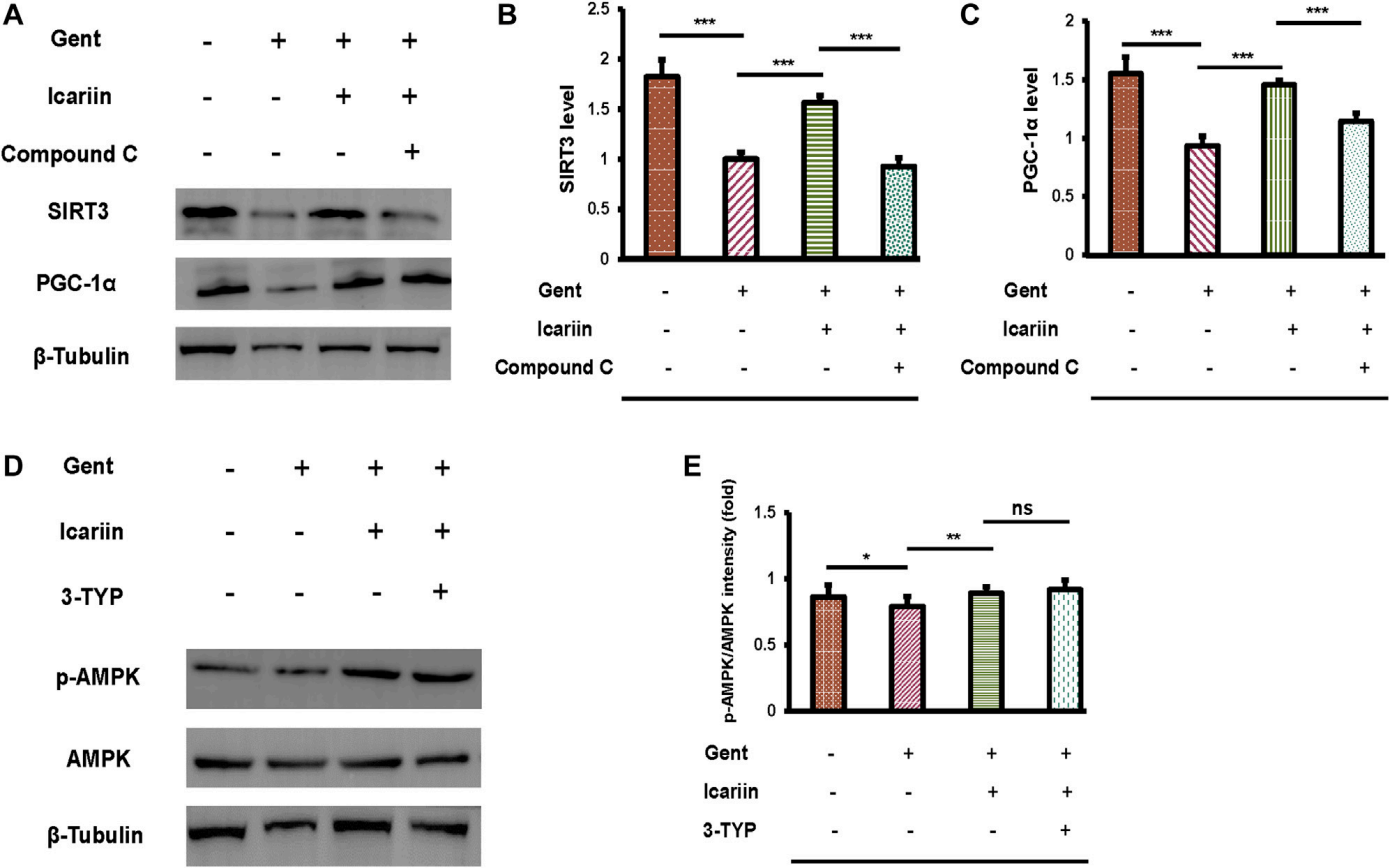
AMPK regulates SIRT3 expression in HEI-OC1 cells. **(A)** We divided HEI-OC1 cells into four groups: control; 500 μM gentamicin treatment for 12 h; 200 μM icariin treatment for 24 h, then 500 μM gentamicin treatment for 12 h; and 10 μM compound C treatment for 2 h, icariin treatment for 24 h, and then gentamicin treatment for 12 h. Western blotting was used to determine the protein expression of SIRT3 and PGC-1α. β-Tubulin was used as a loading control. **(B)** Quantitative analysis of SIRT3 protein expression. **(C)** Quantitative analysis of PGC-1α protein expression. **(D)** HEI-OC1 cells were divided into four groups: control; 500 μM gentamicin treatment for 12 h; 200 μM icariin treatment for 24 h, then 500 μM gentamicin treatment for 12 h; and 50 μM 3-TYP treatment for 2 h, icariin treatment for 24 h, and then gentamicin treatment for 12 h. Western blotting was used to determine the protein expression of p-AMPK and AMPK. β-Tubulin was used as a loading control. **(E)** Quantitative analysis of p-AMPK protein expression. Each experiment was performed three times. Ns, not significant; N = 6 in each group; *p < 0.05; ***p < 0.001; one-way analysis of variance and Tukey’s multiple comparison test.

Our previous experiments have demonstrated that SIRT3 and the AMPK signaling pathway are critical for icariin to protect against gentamicin-induced ototoxicity. Thus, the regulatory relationship between SIRT3 and AMPK requires further investigation (Figures 8A–C). Icariin further enhanced SIRT3 (1.57 ± 0.07 vs. 1.00 ± 0.07) and PGC-1α (1.45 ± 0.04 vs. 0.93 ± 0.08) expression in gentamicin-damaged cells, showing that icariin activated PGC-1α/SIRT3 signaling in gentamicin-damaged cells. We then examined the effect of AMPK depletion on PGC-1α/SIRT3 signaling in gentamicin-damaged cells. Notably, we found that compound C suppressed PGC-1α (1.14 ± 0.07 vs. 1.45 ± 0.04) and SIRT3 (0.93 ± 0.09 vs. 1.57 ± 0.07) expression, suggesting that SIRT3 acts as the downstream target of AMPK signaling in hair cells (Figures 8D,E). To further reveal the mechanisms underlying icariin-induced SIRT3 enhancement, SIRT3 was inhibited by the selective inhibitor 3-TYP. 3-TYP treatment resulted in no change in phosphorylated AMPK (0.92 ± 0.07 vs. 0.89 ± 0.21). Taken together, AMPK signaling activation is crucial for inducing icariin-induced SIRT3 enhancement in gentamicin-damaged HEI-OC1 cells.

## Discussion

Aminoglycoside antibiotics are among the major types of antibiotics widely used in medicine. Today, with the rapid increase in infection rate caused by multidrug-resistant bacteria, aminoglycoside antibiotics have been applied as remaining treatment alternatives (Becker and Cooper, 2013). However, frequent use of aminoglycoside antibiotics has many serious side effects, including irreversible renal toxicity and ototoxicity (Leis et al., 2015; Germovsek et al., 2017). Although aminoglycosides are non-toxic, the redox ability of transition metal ions that interact with aminoglycosides induces ototoxicity. For example, aminoglycoside molecules can decompose (separate from internal coordination sites) and chelate metal ions from biomolecules; the amino nitrogen and deprotonated oxygen of the terminal amino sugar ring participate in the decomposition and chelation of metal ions to produce metal complexes. These metal complexes have redox activity and generate ROS, which cause oxidative damage to hair cells (Guthrie, 2008). If auditory hair cells are damaged, subsequent hearing loss is irreversible, as hair cells are not regenerated in mammals (Chen et al., 2019b; Samarajeewa et al., 2019). In recent decades, extensive progress has been made in restoring auditory function by changing the expression of the specific genes responsible for hair cell differentiation (Zheng and Gao, 2000; Mizutari et al., 2013) or by stem cell therapy (Hao and Li, 2019). However, the short survival time of the new hair cells and lack of normal physiological function remain to be overcome (Chen et al., 2019b). Hence, it is essential to develop novel strategies that can protect auditory hair cells or slow their degradation. Our study extends the functional role of icariin in hearing protection on aminoglycoside antibiotics-induced damage.

The anti-ototoxicity role of icariin has been shown to be dependent on AMPK-SIRT3 signaling activation. In particular, icariin was identified as a potent activator of AMPK-SIRT3 signaling, and AMPK or SIRT3 depletion impaired icariin-induced anti-ototoxicity effects. Icariin is a type of flavonoid with various pharmacological properties, including antioxidative, anti-neuroinflammatory, and anti-apoptotic effects (Jin et al., 2019). Icariin protects against iron overload-induced bone loss by suppressing oxidative stress (Jing et al., 2019). Furthermore, icariin protects cardiomyocytes from ischemia-reperfusion injury by reducing mitochondrial oxidative damage (Wu et al., 2018). In parallel with these existing studies, whether icariin is sensitive to gentamicin induced ototoxicity has become an area of interest. Though not the only one cause, the ROS-dependent mitochondrial damage inducing hair cells apoptosis is a major effect of gentamicin-induced ototoxicity. (Dong et al., 2015; Quan et al., 2015; Kim et al., 2017; Han et al., 2020). In our study, icariin pretreatment significantly improved the cell viability of HEI-OC1 cells injured by gentamicin, inhibited HEI-OC1 cell apoptosis, and reduced endogenous ROS production.

SIRT3 is mainly located in mitochondria and is closely related to oxidative stress (Salvatori et al., 2017), and many studies have focused on its anti-ROS effect on inner hair cells (Brown et al., 2014; Quan et al., 2015; Han et al., 2020). The current study demonstrates that both PGC-1α (SIRT3 promoter) and SIRT3 expression is upregulated in icariin-treated HEI-OC1 cells damaged by gentamicin, indicating that SIRT3 plays an important role in the protective effect of icariin against gentamicin ototoxicity. In addition, further studies showed that the destruction of SIRT3 promoted the increase of apoptosis and endogenous ROS in HEI-OC1 cells, suggesting that the intervention of SIRT3 attenuated the antioxidant effect of icariin. Nevertheless, how icariin enhances SIRT3 expression remains to be determined. Many studies have revealed that the AMPK signaling pathway plays an important role in the pharmacological function of icariin (Han et al., 2015; Li et al., 2017; Chen et al., 2019a). Moreover, AMPK mediates the balance between survival and apoptosis in sensory hair cells under stress (Bodmer and Levano-Huaman, 2017). However, the role of AMPK in gentamicin-induced ototoxicity is still unknown.

AMPK is a highly conserved sensor of low intracellular ATP content that is rapidly activated after almost all mitochondrial stress. When the intracellular ATP/AMP ratio changes, AMPK is activated and phosphorylates downstream targets, shifting metabolism to increased catabolism and decreased synthesis (Herzig and Shaw, 2018). Recent studies have shown that AMPK is involved in the development of many disease models including age-related hearing loss, which is caused by the accumulation of ROS-induced mitochondrial disorder (Su et al., 2019). Consistent with previous studies, this study showed that icariin could significantly increase the expression of AMPK phosphorylation in gentamicin-damaged HEI-OC1 cells. Furthermore, this could inhibit the anti-ototoxicity effect of icariin through selective inhibitor compound C, suggesting that AMPK activation may contribute to the protection of icariin against gentamicin-induced ototoxicity. AMPK has been described to directly affect PGC-1α activity through phosphorylation and induce mitochondrial biogenesis (Palikaras and Tavernarakis, 2014). Our previous study has confirmed that PGC-1α regulates SIRT3 activity during gentamicin-induced ototoxicity (Han et al., 2020). In the present study, with an increasing concentration of icariin, PGC-1α and SIRT3 expression and activity were significantly enhanced, and the ototoxicity caused by gentamicin was also weakened. To further confirm the function of SIRT3 in the protective effect of icariin against gentamicin-induced ototoxicity, we used the selective SIRT3 inhibitor 3-TYP to suppress SIRT3 activity, resulting in increased damage to cells and cochlear explants. The results suggested that SIRT3 may also play a key role in the protective effect of icariin upon gentamicin-induced ototoxicity. Accumulating evidence suggests that AMPK could regulate SIRT3. In previous studies, inhibition of AMPK by compound C markedly suppressed SIRT3 levels. However, SIRT3 siRNA could not significantly change AMPK phosphorylation, showing that SIRT3 was the downstream target of AMPK (Yu et al., 2019). Based on these results, the regulatory activity of the AMPK-SIRT3 signaling pathway in the protective effect of icariin against gentamicin-induced ototoxicity has aroused our interest. In our present study, inhibiting AMPK signaling by compound C significantly suppressed PGC-1α and SIRT3 expression, while SIRT3 depletion by 3-TYP remarkably had no obvious effect on AMPK phosphorylation. Our results suggested that the interplay between AMPK and SIRT3 participated in the inhibitory effect of icariin on ototoxicity, and SIRT3 was convincingly regarded as a critical downstream target of AMPK in gentamicin-induced ototoxicity.

Some findings showed SIRT1 is considered a central regulator of mitochondrial biogenesis because it deacetylates PGC-1α (Nogueiras et al., 2012), which was reported can be upregulated with icariin treatment (Wu et al., 2018). SIRT1 could act as AMPK subtream target to boost the antioxidant defense system at cellular level (Zhang et al., 2017). Although is more weakly affected by 3-TYP compared with SIRT3, SIRT1 remains a potential target of 3-TYP. It is possibly to argue that 3-TYP’s anti-SIRT1 effect also contribute to the obstacle of icariin’s protection on gentamicin-induced ototoxicity. So more experiments are required to determine this possibility. Another significant limitation of our research is the lack of *in vivo* experiments, as we only evaluated icariin activity at the cellular and tissue levels. Therefore, further studies to explore the pharmacological effects of icariin *in vivo* in animal models are required.

## Data Availability

The original contributions presented in the study are included in the article/Supplementary Material, further inquiries can be directed to the corresponding author.
