# Supplementary material for: Icariin Treatment Protects Against Gentamicin-Induced Ototoxicity via Activation of the AMPK-SIRT3 Pathway
Source: Front Pharmacol. 2021 Feb 18;12:620741. doi: 10.3389/fphar.2021.620741 (PMC7930742; doi:10.3389/fphar.2021.620741)
Supplement: Supplementary file 1 [file datasheet1.docx]

Supplementary Material

## Supplementary Figures

**
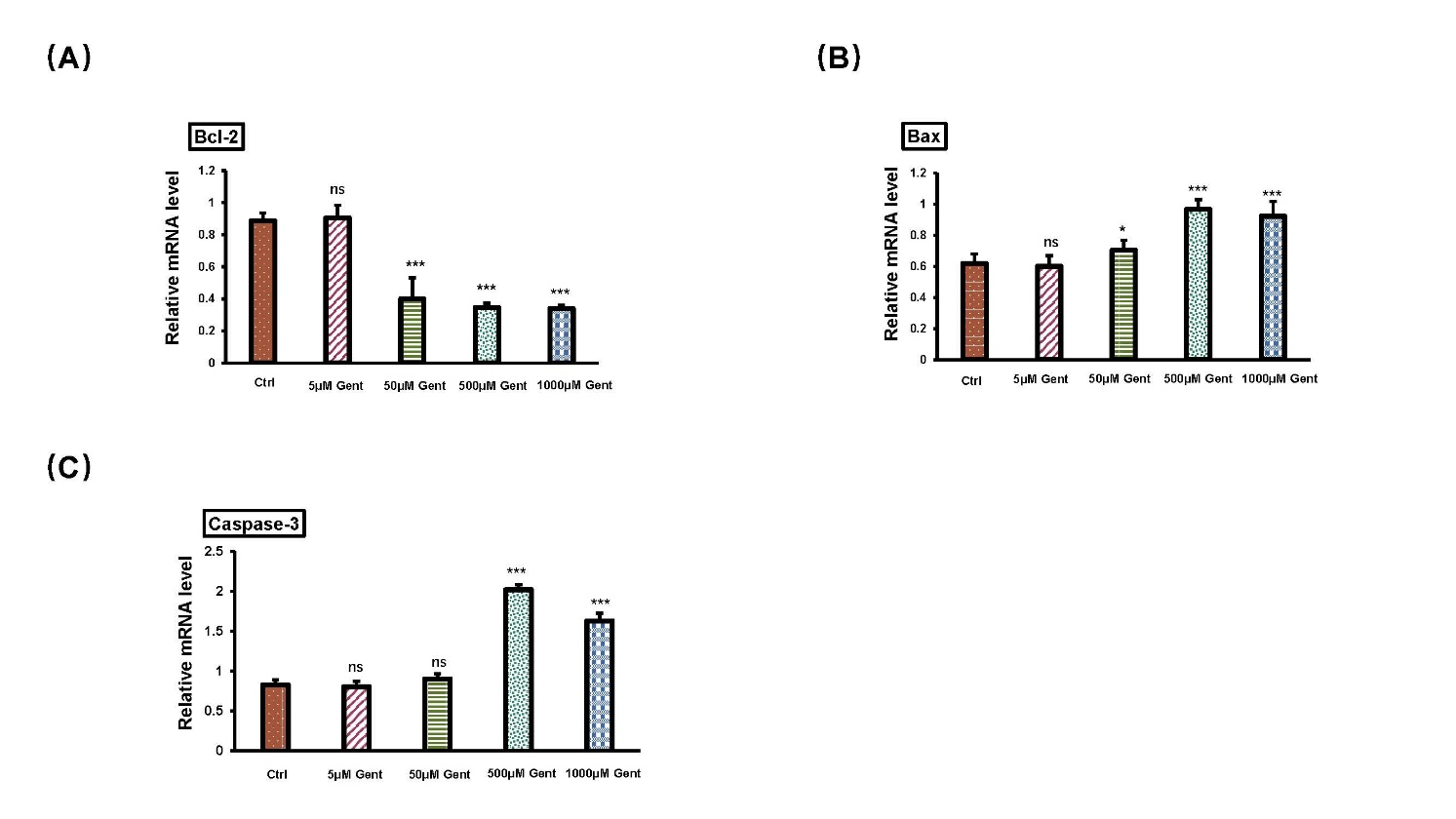
**

**Supplementary Figure 1.** **Gentamicin induces cytotoxicity by activating the mitochondrial apoptotic pathway in HEI-OC1 cells. (A)** mRNA expression of Bcl-2 in gentamicin-treated cells was analyzed by qRT-PCR. **(B)** mRNA expression of Bax in gentamicin-treated cells was analyzed by qRT-PCR. **(C)** mRNA expression of Caspase-3 in gentamicin-treated cells was analyzed by qRT-PCR. Each experiment was performed three times. N=6 in each group. Ns, not significant; *p < 0.05; ***p < 0.001; one-way analysis of variance and Tukey’s multiple-comparison test.


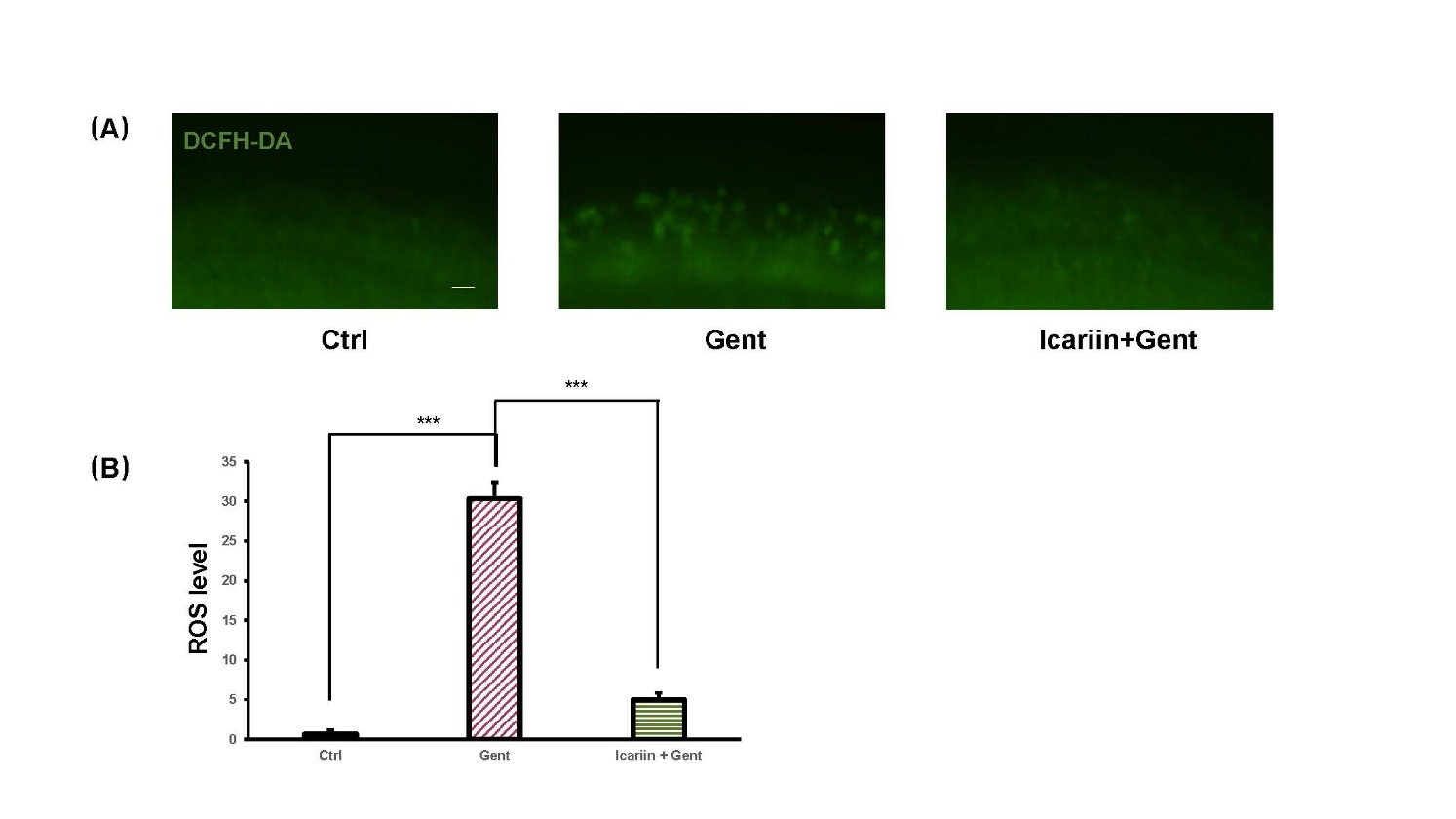


**Supplementary Figure 2. Icariin inhibits reactive oxygen species(ROS) production in cochlear explants. (A)** Cultured cochlear explants were incubated with DCFH-DA working solution (Meilun, China) for 60 minutes, then washed three times with PBS. Photographs were taken by confocal microscopy. **(B)** Quantitative analysis of the number of ROS positive cells in cochlear epithelium cultures. Scale bar = 20μm. Each experiment was repeated thrice. N=6 in each group. Ns, no statistical significance; *p < 0.05, ***p < 0.001; one-way ANOVA followed by Tukey’s multiple-comparison test.


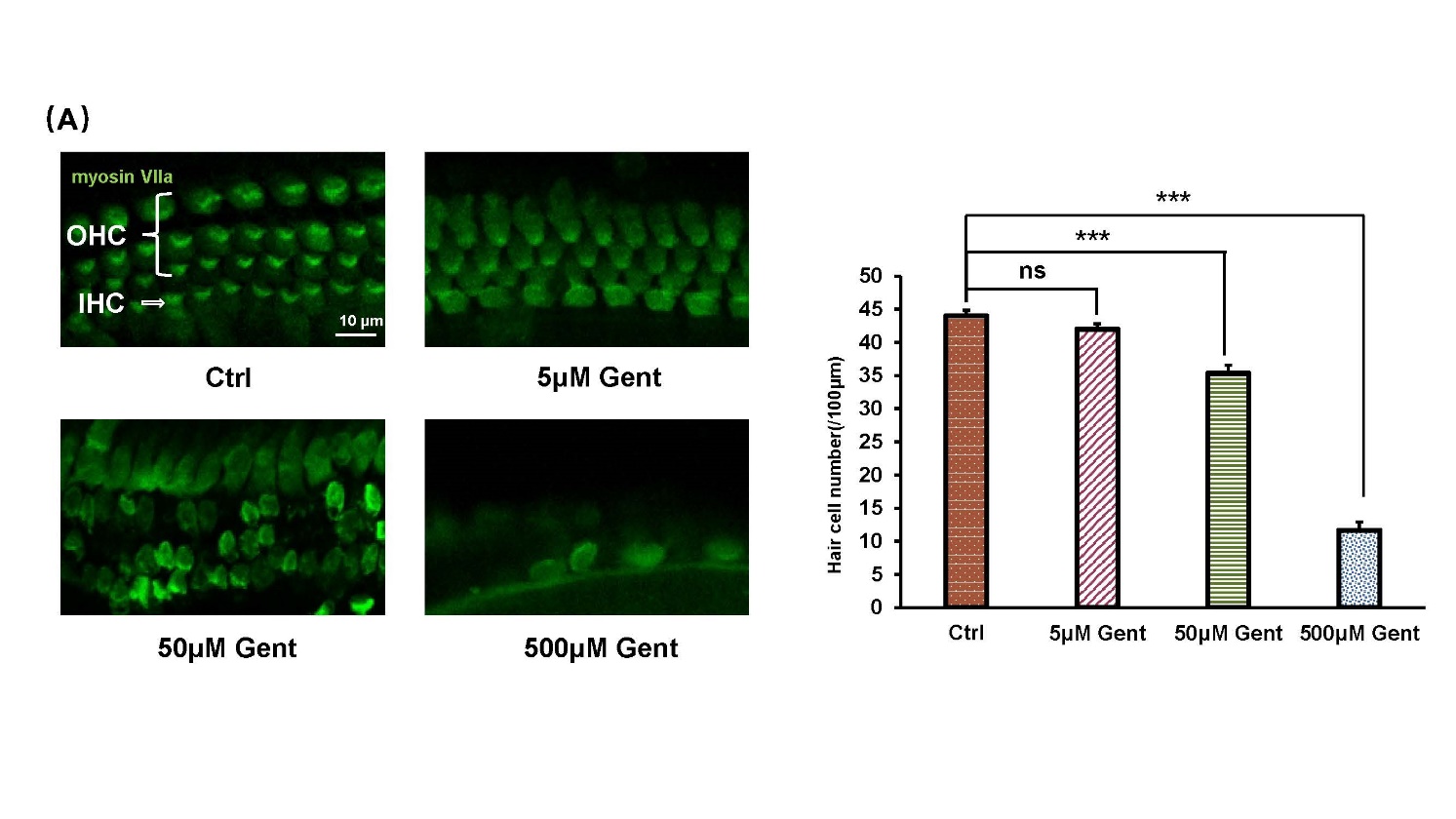


**Supplementary Figure 3. Gentamicin reduces hair cell viability in cultured cochlear explants. (A)** Cultured cochlear explants were treated with increasing concentrations of gentamicin (0, 5, 50, and 500 μM). IHC and OHC were labeled with myosin VIIa (green); representative culture images were obtained by confocal microscopy. Each experiment was performed three times. Scale bar = 10 μm. N=6 in each group. Ns, not significant; *p < 0.05; ***p < 0.001; one-way analysis of variance and Tukey’s multiple-comparison test.


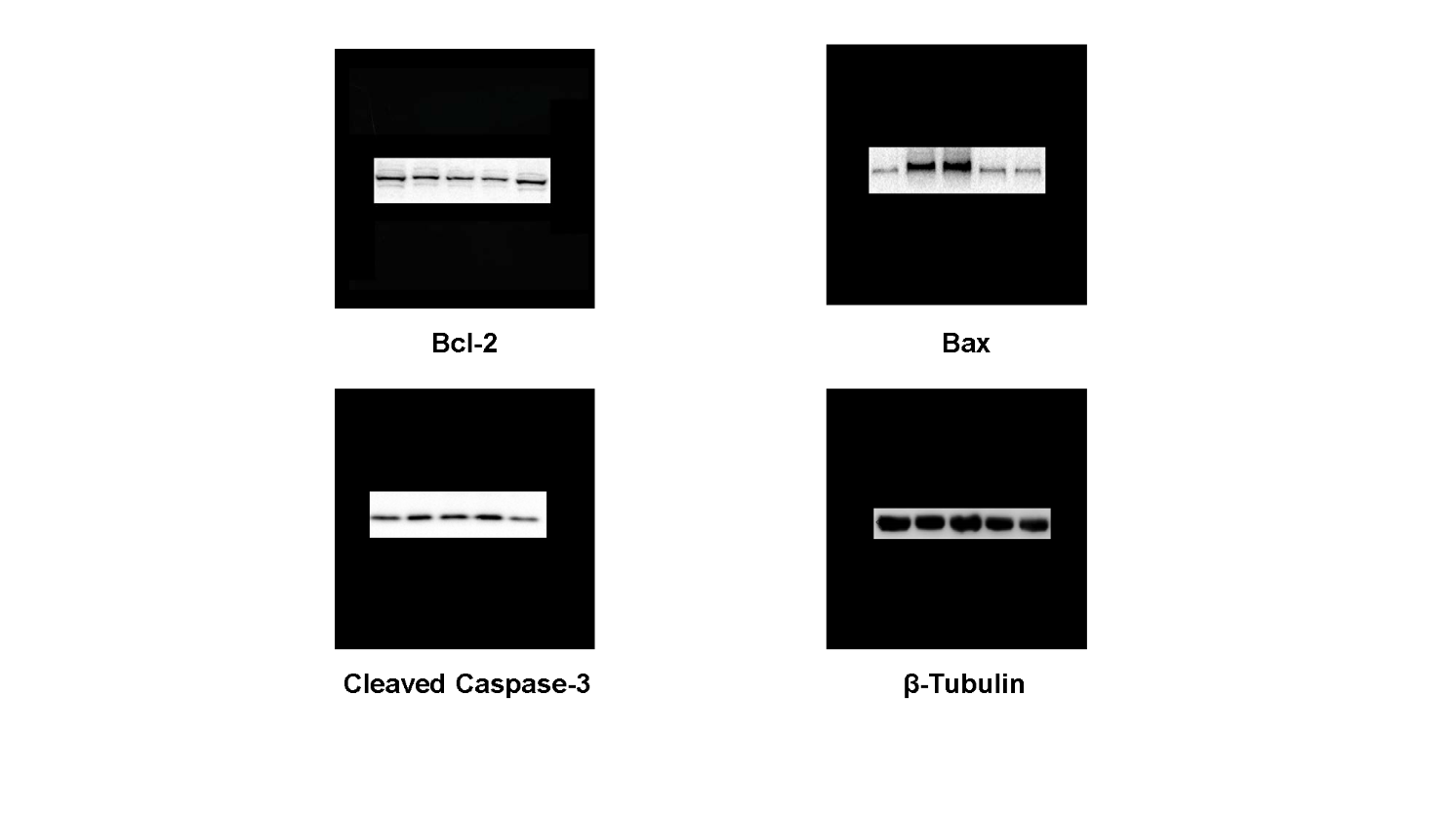
**Supplementary Figure 4. First photograph of Western Blot in Figure 2.**

**
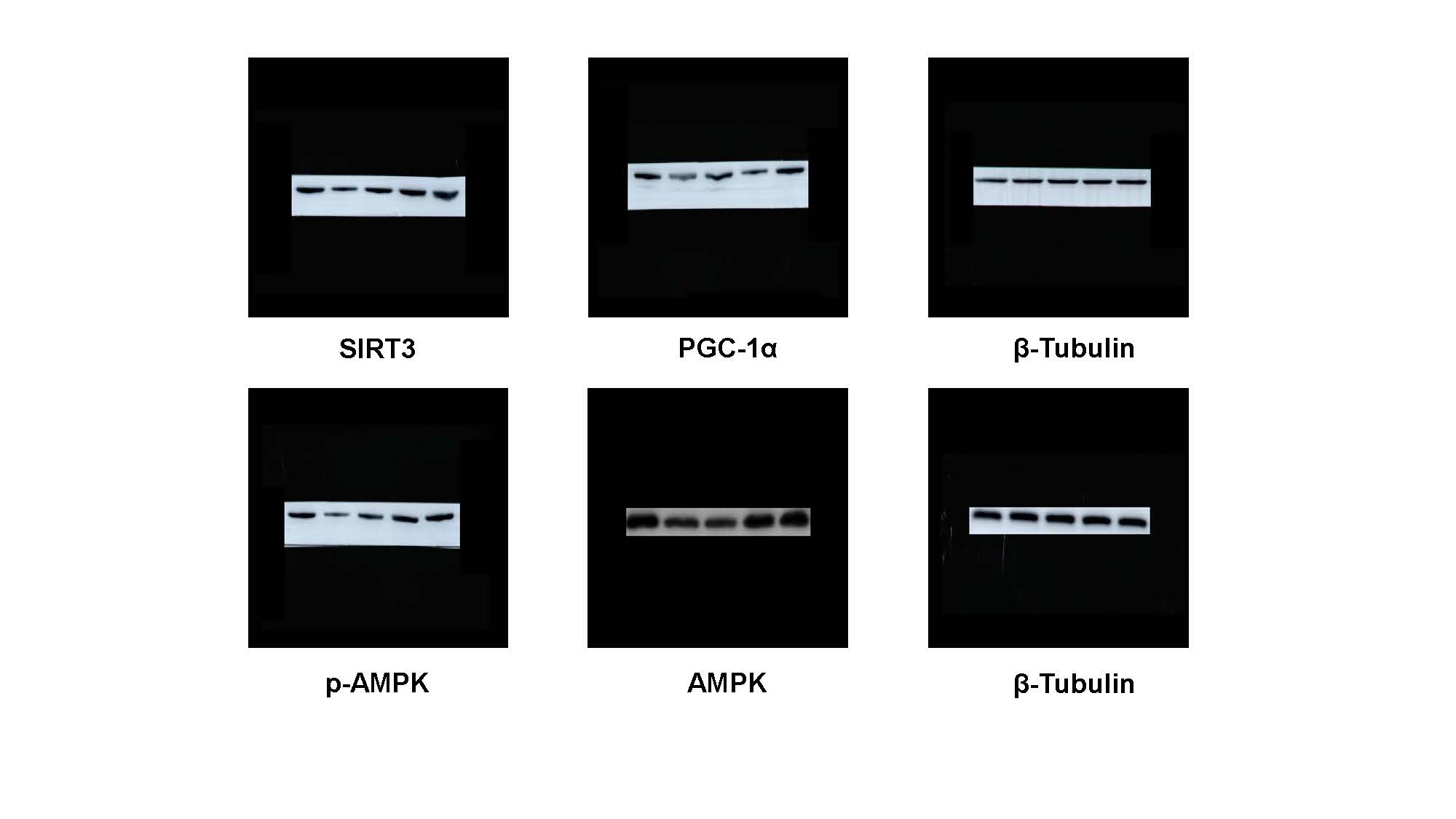
 Supplementary Figure 5. First photograph of Western Blot in Figure 4.
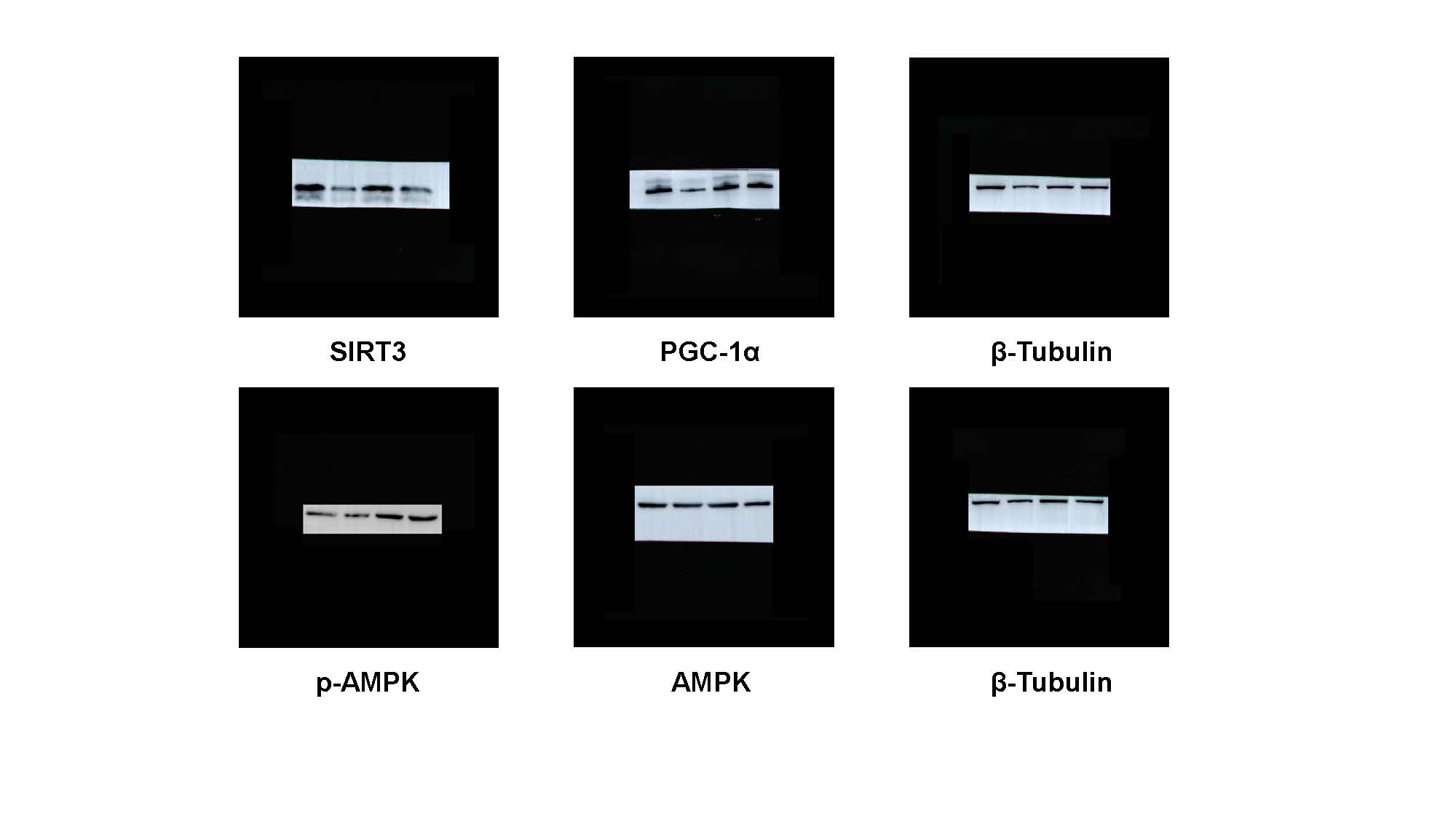
**

**Supplementary Figure 6. First photograph of Western Blot in Figure 8.
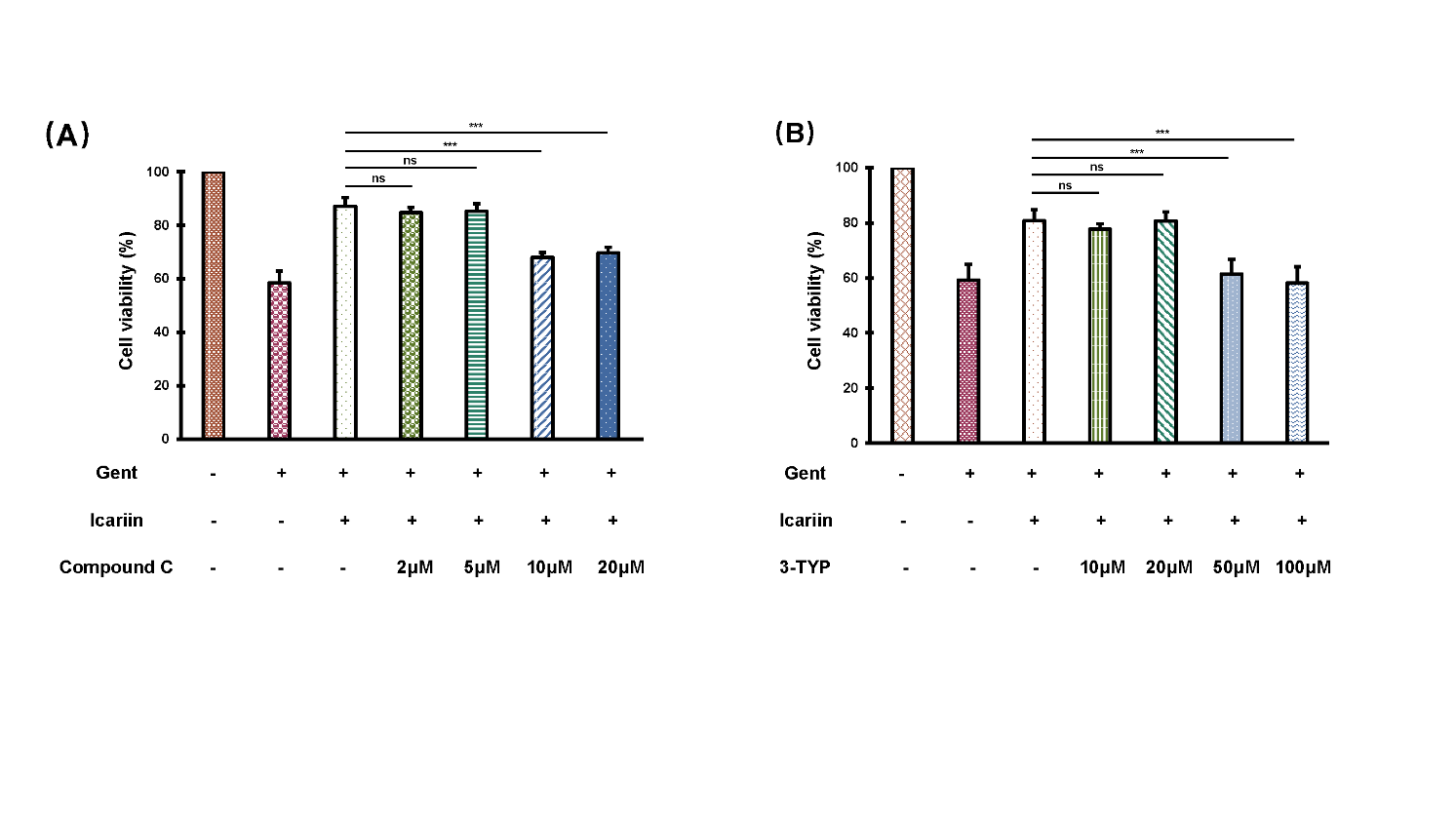
Supplementary Figure 7. Determination of compound C and 3-TYP working concentration. (A)** HEI-OC1 cells were pretreated with increasing concentration of compound C: 0, 2, 5, 10 and 20μM for 2h. Icariin’s protective effect began to decrease significantly when compound C concentration came to 10μM. **(B)** HEI-OC1 cells were pretreated with increasing concentration of 3-TYP: 0, 10, 20, 50 and 100μM for 2h. Icariin’s protective effect began to decrease significantly when 3-TYP concentration came to 50μM. Each experiment was performed three times. N=6 in each group. Ns, not significant; *p < 0.05; ***p < 0.001; one-way analysis of variance and Tukey’s multiple-comparison test.

**
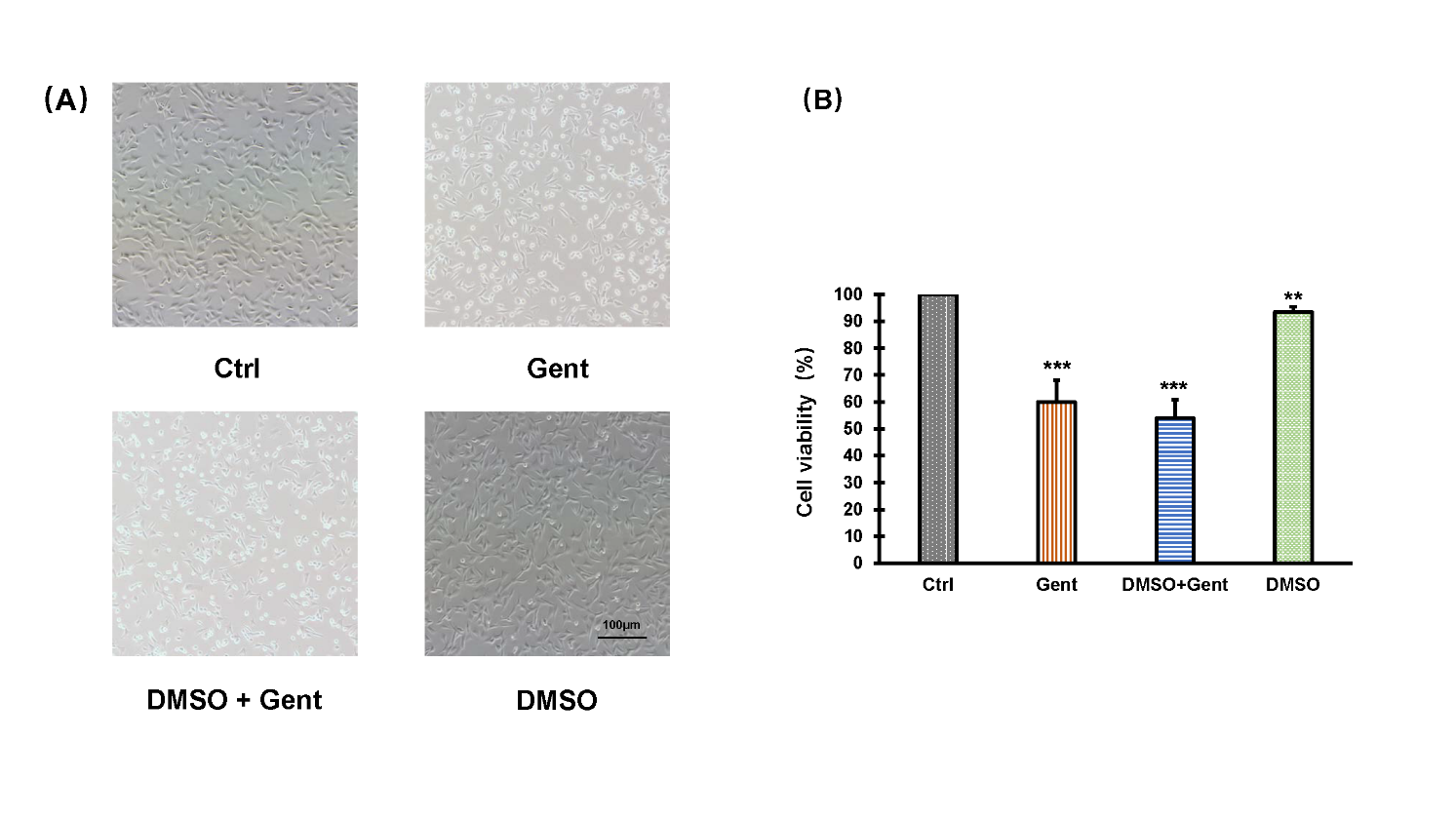
**

**Supplementary Figure 8. Effect of DMSO upon gentamicin-induced cytotoxicity in HEI-OC1 cells. (A)** We divided HEI-OC1 cells into four groups: Control; Gentamicin treatment for 12h; 0.1% DMSO treatment for 24h, then gentamicin treatment for 12h; 0.1% DMSO treatment for 36h. 0.1% DMSO did not show any protective effect upon gentamicin-induced cytotoxicity. Scale bar = 100μm. **(B)** Quantitative analysis of the cell viability in (A). Each experiment was performed three times. N=6 in each group. Ns, not significant; *p < 0.05; ***p < 0.001; one-way analysis of variance and Tukey’s multiple-comparison test.

**
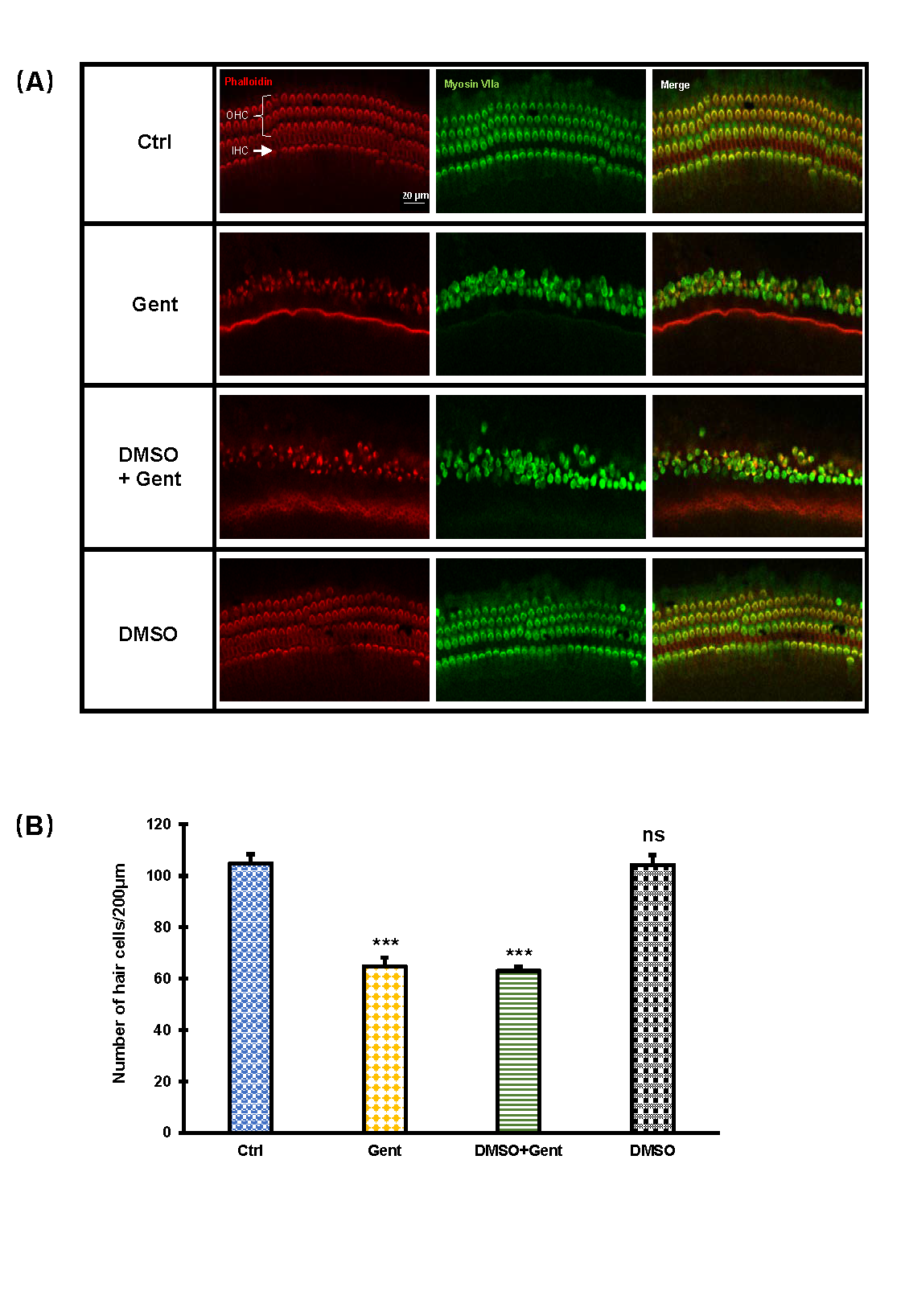
Supplementary Figure 9. Effect of DMSO upon gentamicin-induced cytotoxicity in cochlear explants. (A)** We divided cochlear explants into four groups: Control; Gentamicin treatment for 12h; 0.1% DMSO treatment for 24h, then gentamicin treatment for 12h; 0.1% DMSO treatment for 36h. Samples from the four treatment groups were labeled with Phalloidin (red) and Myosin VIIa (green), which indicated 0.1% DMSO did not show any protective effect upon gentamicin-induced cytotoxicity. **(B)** Quantitative analysis of DMSO’s effect on cochlear explants in (A). Each experiment was performed three times. N=4 in each group. Ns, not significant; *p < 0.05; ***p < 0.001; one-way analysis of variance and Tukey’s multiple-comparison test.
